# The mediating role of empathy in the association between interpersonal communication competence and attitudes toward disability in higher education

**DOI:** 10.1371/journal.pone.0358732

**Published:** 2026-11-06

**Authors:** Şenay Delimehmet Dada, Adile Emel Sardohan Yıldırım

**Affiliations:** 1 Special Education Department, Fatih Faculty of Education, Trabzon University, Trabzon, Turkey; 2 Special Education Department, Faculty of Education, Akdeniz University, Antalya, Turkey; University of Salerno, ITALY

## Abstract

Developing positive attitudes toward people with disabilities is crucial for social equality and integration. This study examines the relationship between university students’ attitudes toward people with disabilities, their empathy levels, and their interpersonal communication competence, with a focus on the mediating role of empathy. Data from 587 students were collected using a cross-sectional survey design and analyzed using correlation analysis and path analysis–based mediation modeling. The findings indicate that higher empathy and communication competence are associated with more positive attitudes and lower levels of prejudice. Empathy partially mediated the association between communication competence and egalitarian attitudes, whereas the indirect association between communication competence and prejudiced attitudes was not statistically significant. In addition, demographic factors such as gender and age were associated with attitudes, with female students reporting more positive views. These results underline the relevance of empathy and communication skills for inclusive attitudes and suggest that higher education institutions may consider policies that strengthen these competencies. Overall, this study contributes to understanding how empathy and communication are associated with disability-related attitudes in higher education.

## Introduction

Communication competence is a set of important skills that enable individuals to establish harmonious and meaningful relationships with each other [1,2]. This competence includes the capacity to interact effectively, appropriately, and successfully in social contexts and is considered an important factor associated with higher-quality social relationships and more efficient interactions [2]. Especially in environments with high social diversity, these skills support social cohesion, inclusion, and social understanding [3,4]. In this context, interpersonal communication plays a key role in the development of social cohesion and inclusion. In higher education institutions in particular, this form of communication is closely associated with more positive attitudes toward people with disabilities and with the adoption of more inclusive approaches [5,6].

Research shows that interpersonal communication, and especially direct contact with people with disabilities, is strongly associated with more positive attitudes toward these individuals [7–12]. The study by Rademaker and colleagues emphasizes that informing typically developing students about disability and supporting this information with constructive contact contributes to the development of inclusive attitudes as well as social participation [12]. In this context, regular and meaningful interactions with people with disabilities have been reported to support positive social interactions and to be associated with reduced prejudice. At the same time, it has been argued that such interventions should be integrated into the education curriculum in order to achieve lasting results.

Not only frequency of contact, but also the demographic characteristics and social position of individuals play an important role in shaping attitudes toward people with disabilities [9,11]. Especially in higher education, factors such as gender, social status, and personal experiences appear to be associated with the formation of attitudes toward people with disabilities, together with the frequency of contact [11,12]. On this topic, Gedik and Toker reported that female students exhibit more positive attitudes toward people with disabilities than male students [13]. Their study also showed that students at private universities reported higher levels of social favorability, whereas students at public universities reported more positive attitudes toward people with disabilities. These findings highlight the associations of social and demographic factors with attitudes toward people with disabilities in higher education. Similarly, Armstrong and colleagues support the view that demographic characteristics such as gender are important factors in the development of these attitudes [7]. While the study by Meyer and colleagues draws attention to the importance of personal experiences and social status for attitudes [9], Keith and colleagues report that individuals’ past direct experiences with people with disabilities are associated with more inclusive attitudes and reduced prejudice [10].

While attitudes shaped by demographic and social factors influence the social experiences of people with disabilities in higher education institutions, the presence of people with disabilities in these institutions is regarded as a reflection of social participation and inclusion beyond individual interactions [9,11,14]. The full participation of people with disabilities in higher education is not limited to the provision of physical accessibility; it is also shaped by social attitudes and interpersonal relations [7,13]. Therefore, while higher education plays an important role in increasing the social participation of people with disabilities, attitudes toward people with disabilities in the higher education environment are seen as one of the most important factors related to the participation of these individuals in educational processes [8,10]. In particular, empathy stands out as a key mediating mechanism associated with more positive attitudes toward people with disabilities [12,15–17].

### Interpersonal communication and empathy

Empathy is the ability of an individual to understand and share the emotional state of another [18]. In educational settings, empathy allows individuals to accept differences and to approach one another with greater understanding [18]. Especially when empathy toward people with disabilities is not well developed, prejudice and misunderstanding toward these individuals may become more common [7]. At this point, interpersonal communication competence plays a major role in improving individuals’ ability to use empathy skills effectively [16]. A study conducted by Han and Park found that empathy training was positively associated with students’ empathy skills, their attitudes toward people with disabilities, and their empathy-based dialogue behaviors [16]. Individuals with higher levels of empathy tend to exhibit more inclusive and positive attitudes in their interactions with people with disabilities [15,17]. Empathy not only enables an individual to understand the difficulties experienced by people with disabilities but is also associated with a greater desire to establish social ties with these individuals. In this context, empathy and regular social interaction stand out as powerful tools for reducing prejudice against people with disabilities and for fostering an inclusive environment. Research has shown that strengthening empathy skills is associated with the development of more positive attitudes toward people with disabilities [16].

When the studies on the subject are examined, various results emerge regarding the positive association of empathy levels and interpersonal communication competencies with attitudes toward people with disabilities in higher education settings. The literature confirms that these two factors play an important role in improving attitudes toward people with disabilities [7,11,16], but it also indicates that comprehensive studies are needed in the higher education context [2,14,19]. In particular, the studies of Meyer and colleagues indicate that social interaction, direct contact, and the participation of people with disabilities in social life are positively associated with attitudes toward people with disabilities [9], while researchers such as Polo Sánchez and Selimović emphasize that empathy levels are decisive in this process [11,18].

Worldwide, disability rates play an important role in the participation of people with disabilities in social life. According to the World Health Organization (WHO), an estimated 1.3 billion people, or about 16% of the global population, experience significant disability [23]. In the United States, more than one in four adults (28.7%) report having some type of disability, and this proportion is higher among older adults [20]. According to data from the Turkish Statistical Institute (TÜİK, formerly the State Institute of Statistics), the disability rate in Turkey was reported as 12.29% in the 2002 Turkey Disability Survey [21]. These figures reflect changes in the definition of disability, increased global awareness, and evolving social attitudes toward disability [20]. Therefore, these data are important for understanding the integration of people with disabilities into society and the level of social participation. Higher education institutions make an important contribution to the social and economic development of people with disabilities [14]. However, the full participation of people with disabilities in higher education should not be limited to physical accessibility; this process should also be supported by social attitudes and interpersonal relationships [7,13]. Higher education institutions have a critical role in fostering more inclusive social attitudes, which are among the most important factors related to the participation of people with disabilities in educational processes [11,12].

### Attitudes toward people with disabilities in higher education in Turkey

In Turkey, attitudes toward people with disabilities are shaped by various forms of prejudice and by limited awareness, both at the societal level and in higher education institutions. Among these shortcomings, inadequate educational policies, social attitudes, and structural barriers stand out. Researchers have reported that Turkish education policies do not adequately respond to the needs of students with disabilities and focus on integration rather than inclusion [22]. Despite legislative initiatives and legal regulations, many policies cannot be effectively implemented in higher education [23]. In addition, students with disabilities at Turkish universities have reported dissatisfaction with universities’ psychosocial environments and the ways in which disability is perceived [24]. Limited awareness about disability among the general population makes this situation even more difficult. The greatest obstacles to the participation of people with disabilities in higher education can be defined as physical access difficulties together with a lack of social acceptance and prejudice. In the literature, empathy levels and interpersonal communication competencies are seen as playing a critical role in improving attitudes toward people with disabilities.

Studies conducted in Turkey also confirm that empathy training is an important tool for improving attitudes toward people with disabilities in higher education [25]. International studies further show that individuals who have received empathy training tend to hold reduced prejudice against people with disabilities and to develop more inclusive attitudes [26]. Empathy is also associated with greater acceptance and more positive attitudes toward people with disabilities in educational settings [15,27–29]. Educators’ attitudes on this issue are critical for successful inclusive education; for example, limited knowledge about inclusive education has been associated with less favorable attitudes toward inclusion [30]. Improving interpersonal communication skills and empathy is associated not only with reduced prejudice against people with disabilities but also with the promotion of social cohesion and inclusion. These findings suggest that empathy training and the development of interpersonal communication skills are fundamental tools for reinforcing supportive and inclusive attitudes toward people with disabilities [11,12].

However, the combined associations among empathy, interpersonal communication competence, and attitudes toward people with disabilities remain insufficiently examined in Turkish higher education. Given the importance of empathy and interpersonal communication skills for attitudes toward people with disabilities, how these skills relate to social attitudes toward people with disabilities should be examined in more depth. This study was conducted to better understand the mediating role of empathy in the association between interpersonal communication competence and attitudes toward people with disabilities in higher education. By addressing this gap in the literature, it aims to provide important findings for the social integration of people with disabilities in higher education.

### Current study

Interpersonal communication competence is one of the main factors that determine the quality of the social ties established between individuals [31]. This competency is of vital importance, especially in environments where individuals with different socio-cultural characteristics come together [7]. In the context of people with disabilities, these communication skills not only support interpersonal harmony but also play a critical role in establishing social acceptance and equality [14]. Indeed, attitudes toward people with disabilities are not limited to individual perceptions; they are directly related to social structures, social policies, and human rights norms [9]. Therefore, improving attitudes toward people with disabilities is an important step toward achieving social equality and justice [11].

Higher education institutions are not only places where academic knowledge is transferred but also important social spaces where individuals develop tolerance and understanding and where social values are shaped. In this context, developing empathy skills and strengthening interpersonal communication competencies have a critical role in positively shaping attitudes toward people with disabilities in higher education [16]. However, in developing countries such as Turkey, the full inclusion of people with disabilities still faces significant challenges. These challenges take various forms, such as physical access barriers, social prejudice, and limited awareness [22]. Education in particular has a central role in the integration of people with disabilities into social life [13].

This study makes an important contribution to the literature by addressing the participation of people with disabilities in higher education in the context of Turkey. It examines the associations of interpersonal communication competence and empathy, alongside social prejudice, with attitudes toward people with disabilities in the context of Turkish higher education. While this may inform future research in other developing-country contexts, it is also valuable for understanding the role of the cultural and socio-economic contexts specific to Turkey. Unlike the Western-oriented studies that are frequently encountered in the literature, this study offers a distinctive perspective on understanding and improving attitudes toward people with disabilities in the context of a developing country such as Turkey. This study conducted in Turkey has the potential to serve as an important reference for future studies on the integration of people with disabilities into society and on the achievement of social equality. Supporting this process may also contribute to education, social justice, and social acceptance.

### Research questions

This study aims to examine the association between interpersonal communication competence and attitudes toward people with disabilities in higher education through the mediating role of empathy. The main questions of the study are as follows:

1. What are the attitudes of higher education students toward people with disabilities?
2. How are demographic factors such as gender, grade level, and age associated with attitudes toward people with disabilities?
3. How are empathy and interpersonal communication competence associated with attitudes toward people with disabilities?
4. Does empathy play a mediating role in the relationship between interpersonal communication competence and attitudes toward people with disabilities?

## Method

### Research model

This study was designed as quantitative research based on a cross-sectional survey design to examine higher education students’ attitudes toward people with disabilities in relation to their empathy levels and interpersonal communication competencies. The main purpose of the study was to determine whether empathy plays a mediating role in the relationship between interpersonal communication competence and attitudes toward people with disabilities. In addition, the associations of demographic variables such as gender, age, and grade level with attitudes toward people with disabilities were examined.

### Study group

The study group consisted of 587 university students. Of the participants, 81.43% (*n* = 478) were female and 18.57% (*n* = 109) were male. The mean age of the participants was 28.31 years (*SD* = 7.45). Among the participants, 2.73% (*n* = 16) were first-year students, 13.46% (*n* = 79) were second-year students, 71.55% (*n* = 420) were third-year students, 12.10% (*n* = 71) were fourth-year students, and 0.17% (*n* = 1) was a fifth-year student. While 96.93% (*n* = 569) of the participating students reported no disability, 2.39% (*n* = 14) reported having a disability, and 0.68% (*n* = 4) did not specify their disability status. All participants were adult university students enrolled in higher education in Türkiye. Participation was voluntary. Students were eligible for inclusion if they were at least 18 years old, provided written informed consent, and completed the data collection instruments.

Data collection was conducted between June 2024 and September 2024. Ethical approval for this study was obtained from the Ethics Committee of Akdeniz University (Decision No. 277, dated 29 May 2024). All procedures were carried out in accordance with the relevant guidelines and regulations and with the 1964 Declaration of Helsinki and its later amendments. Written informed consent was obtained directly from all participants prior to data collection. As all participants were adult university students (aged 18 years and above), no parental or guardian consent was required. The consent form included information about the purpose of the study, the voluntary nature of participation, confidentiality, and the right to withdraw from the study at any time without penalty.

### Measurement tools

#### Attitudes toward Disability in Higher Education Scale (QAD-HE).

This scale was developed to measure university students’ attitudes toward people with disabilities [32]. It consists of 27 items and two sub-dimensions: egalitarian attitudes (11 items) and prejudiced attitudes (16 items). Using a 6-point Likert-type scale, participants rate each item from 1 to 6. Accordingly, the possible score range is 11–66 for the egalitarian attitudes dimension and 16–96 for the prejudiced attitudes dimension. Cronbach's alpha (α) and McDonald's omega (ω) reliability coefficients were examined to determine the reliability of the scores obtained from the Attitudes toward Disability in Higher Education Scale for the data set collected within the scope of this study. The reliability coefficients were α = .85 and ω = .86 for the egalitarian attitudes dimension and α = .82 and ω = .83 for the prejudiced attitudes dimension. As these values exceed  .70, the scores obtained from the scale can be considered reliable.

#### Empathy Assessment Scale (EAS).

The Empathy Assessment Scale is a tool used to measure individuals’ empathy levels [33]. It consists of 13 items and three sub-dimensions: social interaction, cognitive behavior, and emotional recognition. It is answered on a 5-point Likert-type scale (1 = strongly disagree, 5 = strongly agree). Cronbach's alpha (α) and McDonald's omega (ω) reliability coefficients were examined for the data set collected within the scope of this study. For the Empathy Assessment Scale, the coefficients were α = .61 and ω = .62 for the social interaction dimension, α = .61 and ω = .67 for the cognitive behavior dimension, and α = .58 and ω = .60 for the emotional recognition dimension. For the Empathy Assessment Scale total score, α = .81 and ω = .83, which exceed the conventional  .70 threshold. However, the coefficients for the social interaction, cognitive behavior, and emotional recognition sub-dimensions were below this threshold and should be interpreted with caution; therefore, only the Empathy Assessment Scale total score was used in the subsequent analyses.

#### Interpersonal Communication Competence Inventory (ICCI).

The Interpersonal Communication Competence Inventory was developed to measure individuals’ interpersonal communication skills [31]. This inventory includes four sub-dimensions (listening, empathy, expression, and social comfort) and consists of 24 items in total. It is answered on a 5-point Likert-type scale. Cronbach's alpha (α) and McDonald's omega (ω) reliability coefficients were examined for the data set collected within the scope of this study. For the Interpersonal Communication Competence Inventory, the coefficients were α = .85 and ω = .86 for the listening dimension, α = .78 and ω = .78 for the social comfort dimension, α = .64 and ω = .69 for the empathy dimension, and α = .43 and ω = .58 for the expression dimension. For the Interpersonal Communication Competence Inventory total score, α = .85 and ω = .87, which exceed the conventional  .70 threshold. However, the coefficients for the empathy and expression sub-dimensions were below this threshold and should be interpreted with caution; therefore, only the Interpersonal Communication Competence Inventory total score was used in the subsequent analyses.

### Data analysis

Because the scales used did not have established norms, k-means cluster analysis was performed on the scale scores to determine the participants’ levels of attitudes toward people with disabilities, empathy, and interpersonal communication competence in higher education. By examining the means of the clusters obtained, the clusters were labeled as low, moderate, and high. To decide on the appropriate method for the difference analyses, the normality of the scale scores at the levels of the independent variables was examined using skewness and kurtosis values. Because the assumption of normal distribution was not met, nonparametric tests were used. The Mann–Whitney U test was used when the independent variable had two levels (gender), and the Kruskal–Wallis H test was used when the independent variable had more than two levels (grade level). Correlations between the scale scores were examined using Spearman correlation analysis, because at least one of the scale scores was not normally distributed; specifically, the Attitudes toward Disability in Higher Education Scale and Empathy Assessment Scale scores were not normally distributed, whereas the Interpersonal Communication Competence Inventory scores were. Path analysis–based mediation models using observed scale scores were used to examine the mediation model established within the scope of this study. The statistical significance of the indirect associations was tested using bias-corrected bootstrap estimation with 5,000 resamples, and 95% bias-corrected confidence intervals (CIs) were computed; an indirect association was considered statistically significant when its 95% CI did not include zero. The mediation analyses were conducted with the jamovi 2.3.28 program. The anonymized data set underlying all reported analyses is available as Supporting Information (S1 Dataset).

## Results

Cluster analysis was used to classify the participants’ attitudes toward people with disabilities in higher education. The Attitudes toward Disability in Higher Education Scale, which is used to determine attitudes toward people with disabilities in higher education, has two dimensions (egalitarian attitudes and prejudiced attitudes) and no total score is obtained from the scale. The participants’ levels of egalitarian and prejudiced attitudes toward people with disabilities in higher education are presented in Table 1.

**Table 1:** Attitude levels of participants toward people with disabilities in higher education.

		*n*	%	Min.	Max.	*M*	*SD*	Statistics
Egalitarian attitudes	Low	73	12.44	11	44	35.97	7.08	*F*(2, 584) = 1614, *p* < .001
	Moderate	191	32.54	45	57	52.61	3.42	
	High	323	55.03	58	66	62.64	2.31	
Prejudiced attitudes	Low	277	47.19	16	29	23.64	3.58	*F*(2, 584) = 1136, *p* < .001
	Moderate	215	36.63	30	43	35.24	3.79	
	High	95	16.18	44	96	52.24	9.17	

*n* = number of participants; % = percentage; Min. = minimum; Max. = maximum; *M* = mean; *SD* = standard deviation.

As shown in Table 1, based on the cluster analysis for the egalitarian attitudes dimension, 12.44% (*n* = 73) of the participants had a low level of egalitarian attitudes toward people with disabilities in higher education, 32.54% (*n* = 191) had a moderate level, and 55.03% (*n* = 323) had a high level. Based on the cluster analysis for the prejudiced attitudes dimension, 47.19% (*n* = 277) of the participants had a low level of prejudiced attitudes, 36.63% (*n* = 215) had a moderate level, and 16.18% (*n* = 95) had a high level.

Cluster analysis was also used to determine the participants’ empathy levels, using the total score of the Empathy Assessment Scale. The participants’ empathy levels are presented in Table 2.

**Table 2:** Participants’ empathy levels.

Level	*n*	%	Min.	Max.	*M*	*SD*	Statistics
Low	64	10.90	13	45	39.17	6.83	*F*(2, 584) = 1259, *p* < .001
Moderate	242	41.23	46	55	51.69	2.71	
High	281	47.87	56	65	59.74	2.58	

*n* = number of participants; % = percentage; Min. = minimum; Max. = maximum; *M* = mean; *SD* = standard deviation.

As shown in Table 2, 10.90% (*n* = 64) of the participants had a low empathy level, 41.23% (*n* = 242) had a moderate level, and 47.87% (*n* = 281) had a high level.

Cluster analysis was used to determine the participants’ interpersonal communication competence levels, using the total score of the Interpersonal Communication Competence Inventory. The results are presented in Table 3.

**Table 3:** Participants’ levels of interpersonal communication competence.

Level	*n*	%	Min.	Max.	*M*	*SD*	Statistics
Low	85	14.48	19	52	46.52	6.94	*F*(2, 584) = 1512, *p* < .001
Moderate	217	36.97	53	63	58.68	2.98	
High	285	48.55	64	75	68.37	3.16	

*n* = number of participants; % = percentage; Min. = minimum; Max. = maximum; *M* = mean; *SD* = standard deviation.

As shown in Table 3, 14.48% (*n* = 85) of the participants had a low level of interpersonal communication competence, 36.97% (*n* = 217) had a moderate level, and 48.55% (*n* = 285) had a high level.

Participants were asked to rate their level of knowledge about disability from 1 (no knowledge) to 4 (good knowledge). Those who answered 1 or 2 were grouped as having little knowledge, and those who answered 3 or 4 were grouped as having a lot of knowledge. Of the participants, 39.52% (*n* = 232) had little knowledge about disability and 60.48% (*n* = 355) had a lot of knowledge. Participants were also asked to rate the type and frequency of their contact with people with physical and sensory disabilities from 1 (almost never) to 6 (constantly). Those who answered 1, 2, or 3 were grouped as having a low frequency of contact, and those who answered 4, 5, or 6 were grouped as having a high frequency of contact. The results are presented in Table 4.

**Table 4:** Types and frequency of contact with people with physical and sensory disabilities.

Contact type	Frequency, *n* (%)	Mean interaction frequency, *M* (*SD*)
No close acquaintance with someone with a physical disability	245 (41.74%)	3.29 (1.73)
Acquaintance with someone with a physical disability as a classmate	154 (26.24%)	2.55 (1.71)
No close acquaintance with someone with a sensory disability (blind or deaf)	194 (33.05%)	2.88 (1.73)
Acquaintance with someone with a sensory disability as a classmate (blind or deaf)	147 (25.04%)	2.43 (1.72)

*Note. n* = number of participants; *M* = mean; *SD* = standard deviation. The mean interaction frequency is reported for participants with more frequent contact.

As shown in Table 4, 41.74% (*n* = 245) of the participants reported having no close acquaintance with a person with a physical disability, whereas 26.24% (*n* = 154) reported knowing a person with a physical disability as a classmate. In addition, 33.05% (*n* = 194) reported having no close acquaintance with a person with a sensory disability (blindness or deafness), whereas 25.04% (*n* = 147) reported knowing a person with a sensory disability as a classmate.

To decide on the appropriate method for analyzing the participants’ attitudes toward people with disabilities in higher education by gender, the normality assumption was examined first. The Attitudes toward Disability in Higher Education Scale scores were not normally distributed across the levels of the gender variable. Because the gender variable had two levels (female and male) and the normality assumption was not met, the Mann–Whitney U test was used. The results are presented in Table 5.

**Table 5:** Analysis of attitudes toward people with disabilities in higher education by gender.

Attitude dimension	Gender	*n*	*M*	Median	Mean Rank	*U*	*p*
Egalitarian attitudes	Female	478	57.16	60.00	312.11	17396.00	< .001
	Male	109	51.22	53.00	214.60		
Prejudiced attitudes	Female	478	31.43	30.00	280.90	19789.50	< .001
	Male	109	37.28	33.00	351.45		

*n* = number of participants; *M* = mean; *U* = Mann–Whitney U statistic; *p* = significance value.

As shown in Table 5, there was a statistically significant difference (*p* < .001) in egalitarian attitudes by gender. The mean egalitarian attitudes score of female participants (*M* = 57.16) was higher than that of male participants (*M* = 51.22); accordingly, female participants reported higher egalitarian attitudes toward people with disabilities in higher education than male participants. There was also a statistically significant difference (*p* < .001) in prejudiced attitudes by gender. The mean prejudiced attitudes score of male participants (*M* = 37.28) was higher than that of female participants (*M* = 31.43); accordingly, male participants reported higher prejudiced attitudes toward people with disabilities in higher education than female participants.

To decide on the appropriate method for examining the participants’ attitudes toward people with disabilities in higher education by grade level, the normality assumption was examined first. The Attitudes toward Disability in Higher Education Scale scores were not normally distributed across the levels of the grade-level variable. Because the grade-level variable had more than two levels (1st–2nd year, 3rd year, and 4th–5th year) and the normality assumption was not met, the Kruskal–Wallis H test was used. The results are presented in Table 6.

**Table 6:** Analysis of attitudes toward people with disabilities in higher education by grade level.

Attitude dimension	Grade level	*n*	*M*	Median	Mean Rank	χ² (2)	*p*
Egalitarian attitudes	1st–2nd year	95	53.38	56.00	263.70	5.530	.063
	3rd year	420	56.72	59.00	304.20		
	4th–5th year	72	55.76	57.50	274.46		
Prejudiced attitudes	1st–2nd year	95	34.42	32.00	321.21	2.943	.230
	3rd year	420	31.96	30.00	288.26		
	4th–5th year	72	33.22	28.00	291.56		

*n* = number of participants; *M* = mean; χ²(2) = Kruskal–Wallis H test statistic with 2 degrees of freedom; *p* = significance value.

As shown in Table 6, there was no statistically significant difference (*p* > .05) in either the egalitarian attitudes dimension or the prejudiced attitudes dimension by grade level. In other words, both egalitarian and prejudiced attitudes toward people with disabilities in higher education were similar regardless of the participants’ grade level.

To decide on the appropriate method for examining the relationship between the participants’ ages and their attitudes toward people with disabilities in higher education, the normality assumption was examined first. Because the Attitudes toward Disability in Higher Education Scale scores were not normally distributed, Spearman correlation analysis was used. The results are presented in Table 7.

**Table 7:** Relationship between participants’ ages and their attitudes toward people with disabilities in higher education.

		Egalitarian attitudes	Prejudiced attitudes
Age	*r*	.26*	−.07

*r* = Spearman rank-order correlation coefficient. *p <  .01.

As shown in Table 7, there was a positive, weak (*r* = .26), and statistically significant (*p* < .01) relationship between the participants’ age and their egalitarian attitudes toward people with disabilities in higher education. Accordingly, higher participant age was associated with slightly higher egalitarian attitudes. There was no statistically significant relationship between the participants’ age and their prejudiced attitudes (*p* > .05).

Spearman correlation analysis was used to examine the relationships among attitudes toward people with disabilities in higher education, empathy levels, and interpersonal communication competence, because the normality assumption was not met. The results are presented in Table 8.

**Table 8:** Relationships among attitudes toward people with disabilities in higher education, empathy levels, and interpersonal communication competence.

Variable	1	2	3	4
1. Egalitarian attitudes	—			
2. Prejudiced attitudes	−.32*	—		
3. Empathy Assessment Scale	.38*	−.18*	—	
4. Interpersonal Communication Competence	.39*	−.31*	.43*	—

Values are Spearman correlation coefficients. 1 = egalitarian attitudes; 2 = prejudiced attitudes; 3 = Empathy Assessment Scale total score; 4 = Interpersonal Communication Competence Inventory total score. *p < .01.

As shown in Table 8, there were positive, moderate (*r* = .38 and  .39, respectively), and statistically significant (*p* < .01) relationships between egalitarian attitudes toward people with disabilities in higher education and both empathy levels and interpersonal communication competence. Accordingly, higher empathy levels and higher interpersonal communication competence were associated with higher egalitarian attitudes. There was a negative, low (*r* = −.18), and statistically significant (*p* < .01) relationship between prejudiced attitudes and empathy levels; accordingly, higher empathy levels were associated with slightly lower prejudiced attitudes. There was a negative, moderate (*r* = −.31), and statistically significant (*p* < .01) relationship between prejudiced attitudes and interpersonal communication competence; accordingly, higher interpersonal communication competence was associated with lower prejudiced attitudes. Finally, there was a positive, moderate (*r* = .43), and statistically significant (*p* < .01) relationship between empathy levels and interpersonal communication competence.

The mediating role of empathy in the statistical association between the participants’ interpersonal communication competence and their attitudes toward people with disabilities in higher education was examined using path analysis–based mediation models with observed scale scores. Separate models were established for the two sub-dimensions of the Attitudes toward Disability in Higher Education Scale. The results are presented in Table 9, and the corresponding path diagrams are presented in Fig 1.

**Table 9:** Mediating role of empathy in the association between interpersonal communication competence and attitudes toward people with disabilities in higher education.

Pathway	Path coefficient (*B*)	Std. path coefficient (β)	*p*	Bootstrap 95% CI (*B*)
**Model 1**				
Interpersonal communication competence → Egalitarian attitudes (total association, no mediator)	0.54	.47	< .05	[0.46, 0.62]
Interpersonal communication competence → Empathy (direct association)	0.44	.51	< .05	[0.38, 0.50]
Empathy → Egalitarian attitudes (direct association)	0.36	.27	< .05	[0.26, 0.46]
Interpersonal communication competence → Egalitarian attitudes (direct association)	0.38	.33	< .05	[0.30, 0.46]
Interpersonal communication competence → Empathy → Egalitarian attitudes (indirect association through empathy)	0.16	.14	< .05	[0.09, 0.24]
**Model 2**				
Interpersonal communication competence → Prejudiced attitudes (total association, no mediator)	−0.48	−.35	< .05	[−0.58, −0.38]
Interpersonal communication competence → Empathy (direct association)	0.44	.51	< .05	[0.38, 0.50]
Empathy → Prejudiced attitudes (direct association)	−0.13	−.08	< .05	[−0.25, −0.01]
Interpersonal communication competence → Prejudiced attitudes (direct association)	−0.42	−.31	< .05	[−0.52, −0.32]
Interpersonal communication competence → Empathy → Prejudiced attitudes (indirect association through empathy)	−0.06	−.04	.064	[−0.13, 0.01]

Path coefficients represent statistical associations estimated from cross-sectional data and are not interpreted as causal effects. The total association is the association without the mediator; the direct and indirect associations were estimated with empathy as the mediator. The statistical significance of the indirect associations was tested using bias-corrected bootstrapping with 5,000 resamples; an association was considered statistically significant when its 95% CI did not include zero. *B* = unstandardized coefficient; β = standardized coefficient; CI = confidence interval.

**Fig 1:**
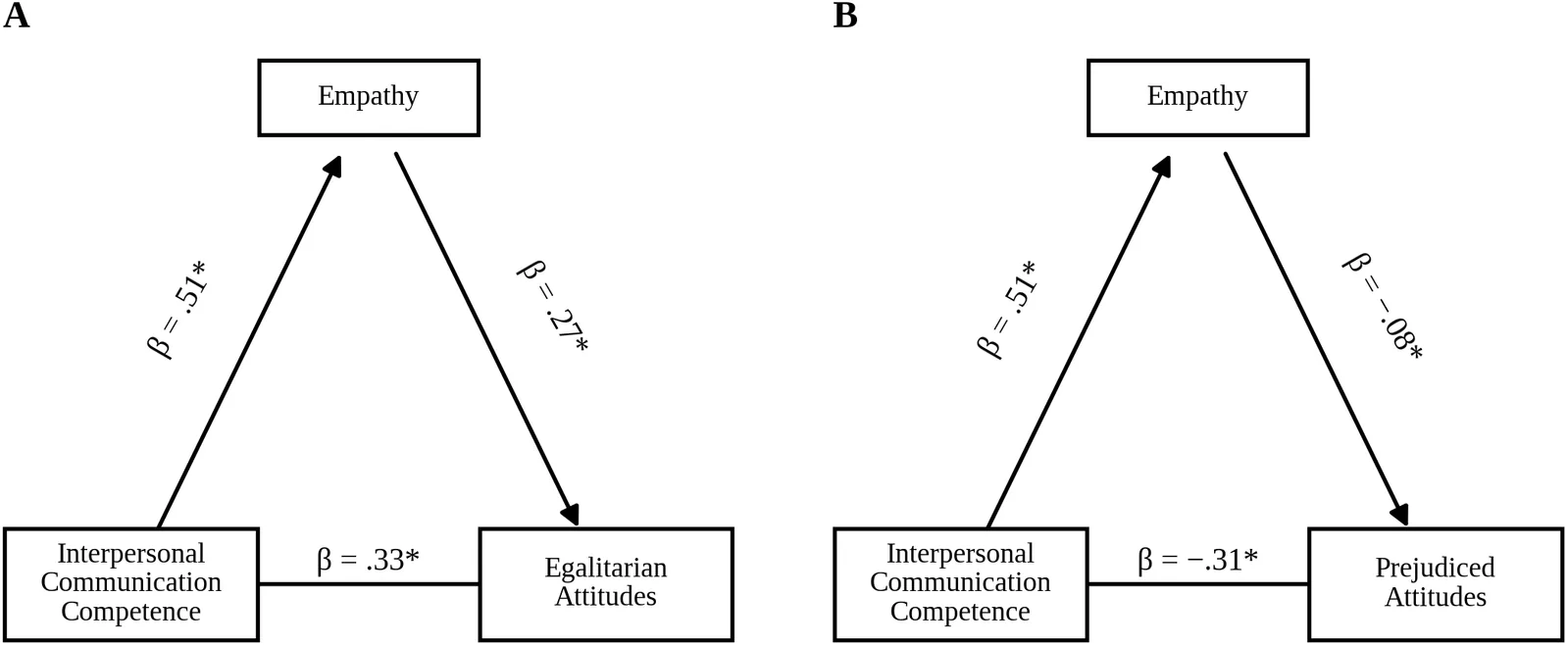
Path diagrams of the statistical associations between interpersonal communication competence, empathy, and attitudes toward people with disabilities in higher education (standardized coefficients). Panel A shows the model for egalitarian attitudes and Panel B the model for prejudiced attitudes. Values are standardized path coefficients (β) estimated from cross-sectional data and represent statistical associations, not causal effects. The indirect association through empathy was β = .14 (Panel A) and β = −.04, p = .064 (Panel B); the total association without the mediator was β = .47 (Panel A) and β = −.35 (Panel B). *p < .05.

As shown in Table 9, the standardized path coefficient from interpersonal communication competence to egalitarian attitudes was β = .47, and this association was statistically significant (*p* < .05). In other words, a one standard deviation higher interpersonal communication competence score was associated with a 0.47 standard deviation higher egalitarian attitudes score. When empathy was included in the model as a mediator, the standardized path coefficient from interpersonal communication competence to empathy (β = .51) and the standardized path coefficient from empathy to egalitarian attitudes (β = .27) were both statistically significant (*p* < .05). With empathy in the model, the standardized path coefficient from interpersonal communication competence to egalitarian attitudes was β = .33 and remained statistically significant (*p* < .05). In addition, the indirect association from interpersonal communication competence to egalitarian attitudes through empathy was statistically significant (*B* = 0.16, β = .14; 95% CI [0.09, 0.24], excluding zero; *p* < .05). Because the association between interpersonal communication competence and egalitarian attitudes remained significant after empathy was included, empathy can be said to have a partial mediating role in the association between interpersonal communication competence and egalitarian attitudes toward people with disabilities in higher education.

As also shown in Table 9, the standardized path coefficient from interpersonal communication competence to prejudiced attitudes was β = −.35, and this association was statistically significant (*p* < .05). In other words, a one standard deviation higher interpersonal communication competence score was associated with a 0.35 standard deviation lower prejudiced attitudes score. When empathy was included in the model as a mediator, the standardized path coefficient from interpersonal communication competence to empathy (β = .51) and the standardized path coefficient from empathy to prejudiced attitudes (β = −.08) were both statistically significant (*p* < .05). With empathy in the model, the standardized path coefficient from interpersonal communication competence to prejudiced attitudes was β = −.31 and remained statistically significant (*p* < .05). However, the indirect association from interpersonal communication competence to prejudiced attitudes through empathy was not statistically significant (*B* = −0.06, β = −.04; 95% CI [−0.13, 0.01], including zero; *p* = .064). Because this indirect association was not significant, empathy does not have a mediating role in the association between interpersonal communication competence and prejudiced attitudes toward people with disabilities in higher education.

## Discussion and conclusion

This study presents important findings by examining the associations of empathy levels and interpersonal communication competencies with attitudes toward people with disabilities. The results revealed that higher empathy levels and interpersonal communication competencies were associated with more egalitarian attitudes toward people with disabilities and with lower prejudiced attitudes. These findings are consistent with the literature on the role of social interaction in shaping attitudes and highlight the multidimensional association of empathy and communication skills with social acceptance [11,16,18].

One of the most notable findings was that empathy partially mediated the association between interpersonal communication competence and egalitarian attitudes. It is consistent with previous studies that empathy-building programs can be effective in shaping individuals’ attitudes toward people with disabilities [16,17]. This study contributes to the literature by showing that empathy is associated with the pathway between interpersonal communication competence and egalitarian attitudes. This suggests that empathy is associated with more positive attitudes, potentially through the quality of social interaction [9].

In addition, the direct association between interpersonal communication competence and egalitarian attitudes is one of the notable findings of the study. According to Spitzberg and Cupach's model of interpersonal communication competence, effective communication skills enable individuals to experience social contexts in a meaningful way [2]. In this framework, the findings indicate that effective interpersonal communication skills are associated with more positive attitudes toward people with disabilities. Bossaert and colleagues reported that more positive attitudes are associated not only with individual characteristics but also with the quality of contact with people with disabilities [3].

Another prominent finding is the association of demographic differences with attitudes toward people with disabilities. Female participants exhibited more egalitarian attitudes than male participants, which is consistent with the study of Gedik and Toker [13]. This difference may be related to broader patterns in the literature linking gender with empathic tendencies, although the present study did not directly test this mechanism. The positive association of age with egalitarian attitudes is also noteworthy; a plausible explanation is that social experience and empathic skills tend to be greater at older ages [14]. However, the absence of a significant difference between grade levels leaves the role of the educational process in these attitudes open to question. It should also be noted that the sample was heterogeneous with respect to age and year of study, with third-year students constituting the large majority of participants; this heterogeneity may partly account for the demographic associations observed and should be considered when comparing these findings with those from more homogeneous samples.

Findings in the literature show that social contact with people with disabilities is very important in shaping attitudes [12]. The studies of Polo Sánchez and Selimović also present findings supporting the association between social contact and positive attitudes [11,18]. However, the association of the quality and continuity of this contact with attitudes needs to be examined in more detail.

In the Turkish context, the association of structural and social shortcomings with the development of attitudes toward people with disabilities is clearly evident. The study of Sakız and Woods revealed the lack of inclusive policies in higher education institutions in Turkey and how this is associated with greater barriers to social interaction with people with disabilities [23]. Studies also indicate that people with disabilities in Turkey face substantial barriers to accessing and participating in higher education [34,35]. This suggests that social prejudice and limited awareness are as influential as physical accessibility barriers. Addressing physical accessibility may support social acceptance but is unlikely to be sufficient on its own. It is therefore important to develop social programs that support empathy and interpersonal communication skills.

In conclusion, the findings of this study point to the potential value of comprehensive policies and programs that support more inclusive and positive attitudes toward people with disabilities in the Turkish context. Such policies should aim not only to increase physical accessibility but also to raise social awareness, strengthen empathy skills, and enhance interpersonal communication competencies. In this way, people with disabilities may be included in higher education in a more inclusive and equitable manner. This process may also contribute to strengthening the principles of social justice and equality.

### Limitations and recommendations

This study has several limitations. First, a cross-sectional survey design was used, and this design is suited to describing the current situation rather than establishing cause-and-effect relationships. A longitudinal research design would be more useful for understanding how the associations of empathy and interpersonal communication competence with attitudes change and are sustained over time. In addition, only a sample of university students in Turkey was used, which limits the generalizability of the findings to different cultures and socio-economic contexts. The use of only self-report scales may increase the risk of social desirability bias and may not fully reflect the participants’ actual attitudes. Furthermore, the sample was heterogeneous with respect to age (*M* = 28.31, *SD* = 7.45) and year of study, with third-year students constituting 71.55% of participants. This heterogeneity may have influenced the observed associations and limits the generalizability of the findings; future studies using more homogeneous or stratified samples, or statistically controlling for age and year of study, would help clarify the extent to which these characteristics shape the associations reported here.

Another limitation is that attitudes toward people with disabilities were analyzed at the individual level. However, the role of macro-level factors such as social structure, legal regulations, and social norms in these attitudes should also be taken into consideration. For example, the role of higher education institutions’ practices, ranging from physical accessibility to opportunities for social interaction, in the development of such attitudes should be examined. In addition, the finding that the indirect association of empathy with prejudiced attitudes was not statistically significant points to the more complex nature of these attitudes and suggests the need for more comprehensive analyses.

Despite these limitations, several recommendations can be made in light of the findings. First, longitudinal studies should be conducted to understand the associations of empathy and interpersonal communication competencies with attitudes in more depth. Such studies could reveal how these skills develop and how they are associated with attitudes over time. Conducting studies with larger samples that include different age groups, occupational fields, and cultural contexts could increase the generalizability of the findings. In addition, including people with disabilities in the research may provide unique information based on their experiences.

Multimethod approaches should be adopted to reduce social desirability bias and obtain more reliable data. In addition to self-report scales, the use of observational and qualitative methods would make it possible to evaluate individuals’ attitudes toward people with disabilities in more depth. Furthermore, studies that consider social policies and legal regulations should be conducted in order to understand how attitudes addressed at the individual level relate to the social level.

Finally, it is suggested that higher education institutions establish training programs that develop empathy skills and reduce prejudice against people with disabilities. These programs should focus not only on theoretical knowledge but also include practical applications and direct interaction with people with disabilities. Beyond providing physical accessibility, higher education institutions need to develop inclusive policies that encourage social interaction with people with disabilities. Such initiatives would not only support more positive interpersonal attitudes but also contribute to strengthening social equality and social justice.

## Supporting information

S1 Dataset Anonymized dataset. A De-identified scale item responses and total scores, together with aggregate and analysis-specific demographic frequency tables, supporting the analyses and tables reported in the manuscript.
(XLSX)
